# A study of dynamic hand orthosis combined with unilateral task-oriented training in subacute stroke: A functional near-infrared spectroscopy case series

**DOI:** 10.3389/fneur.2022.907186

**Published:** 2022-08-11

**Authors:** ChaoJinZi Li, Yih Wong, Birgitta Langhammer, FuBiao Huang, XiaoXia Du, YunLei Wang, HaoJie Zhang, Tong Zhang

**Affiliations:** ^1^School of Rehabilitation Medicine, Capital Medical University, Beijing, China; ^2^Department of Neurological Rehabilitation, Beijing Bo'ai Hospital, China Rehabilitation Research Center, Beijing, China; ^3^Department of Research, Sunnaas Rehabilitation Hospital, Bjornemyr, Norway; ^4^Faculty of Medicine, Institute of Clinical Medicine, University of Oslo, Oslo, Norway; ^5^Department of Physiotherapy, Faculty of Health Science, OsloMet-Oslo Metropolitan University, Oslo, Norway; ^6^Department of Occupational Therapy, Beijing Bo'ai Hospital, China Rehabilitation Research Center, Beijing, China

**Keywords:** stroke, task-oriented therapy, orthosis, upper extremity, neurorehabilitation, near-infrared spectroscopy

## Abstract

**Background:**

Motor dysfunction in the upper extremities after stroke prohibits people with stroke from being independent in daily living. The application of fNIRS to explore brain activity under rehabilitation intervention is a research focus on neurorehabilitation.

**Objective:**

The purpose of this study was to explore, using a grip-release ring motor task, the activated changes of regions of interest and changes in motor function utilizing fNIRS technology and test scales on persons with stroke who received unilateral task-oriented therapy with a hand orthosis in the early subacute stroke period before and after intervention. The study aimed to find a sensitive motor task and region of interest first, then to evaluate the feasibility and mechanism of this rehabilitation method by utilizing fNIRS technology in the next randomized controlled trial.

**Methods:**

In this case series, eight right-handed, right hemiplegia subacute stroke persons (6 males,2 females from age 47 to 72) were enrolled. They received 30 min of unilateral task-oriented therapy without orthosis and 30 min of unilateral task-oriented therapy with orthosis (5 days/week) for 4 weeks. Activated channel numbers and beta values based on oxygenated hemoglobin concentration change using a grip-release ring motor task were estimated with fNIRS. Clinical outcome measures, including grip strength evaluation, action research arm test, and Fugl-Meyer assessment of the arm, were evaluated at the same time.

**Results:**

Individual activation analysis showed that, after intervention, Subjects 1, 2, 6, 7, and 8 had the maximum mean beta value located in the left premotor cortex, while Subjects 4 and 5 had the maximum mean beta value located in the left sensorimotor cortex. The activation analysis of Subject 3 showed the maximum mean beta value located in the right premotor cortex. Deactivations of left sensorimotor cortex, left premotor cortex, and bilateral prefrontal cortex were observed after intervention which were different from other cases. Group activation analysis showed that bilateral cerebral hemispheres were activated in all eight participants, with right hemisphere and right supplementary motor cortex activated dominantly. After the intervention, the activation of bilateral hemispheres decreased but in different brain regions; there was a trend that the activation intensity of left sensorimotor cortex, right premotor cortex, and right prefrontal cortex decreased while activation intensity of left premotor cortex and left prefrontal cortex increased. Each participant demonstrated improvements in all the clinical test scales after intervention.

**Conclusions:**

Left premotor cortex, left sensorimotor cortex, and right supplementary motor cortex may be the primary regions of interest. Grasp-release ring task was not appropriate to achieve our fNIRS research objective and a more sensitive motor task or more sensitive evaluating indicator should be used in further studies.

## Introduction

Persons with stroke may experience a high rate of disability (1, 2). In the acute stage of stroke, two-thirds may experience upper limb and hand dysfunction 3 months after stroke onset; 50% still experience disability concerning arm-hand performance (3, 4). Many persons with stroke (30–66%) experience reduced motor function in the upper extremities 72 months after debut (4, 5).Clinically, it is very important to explore methods to enhance the upper limb motor function, since it makes it challenging for the persons with stroke to engage in activities throughout the day and to perceive quality of life (6, 7).In order to select effective rehabilitation treatment, the mechanisms and effectiveness of the interventions must be known.

An important aim of rehabilitation is to promote neuroplasticity within the central nervous system and to compensate or regain the ability of daily practice through learning, as neurological function re-organization occurs in response to experience. The use of task-oriented training, focusing on the practice of meaningful tasks aimed at acquiring a skill, rather than simple repetition of a movement (8), was supported by growing evidence (9, 10). Assistive Technologies (ATs), defined as “electrical or mechanical devices designed to help people recover movement” (11), such as dynamic orthoses fitted to the affected arm-hand to facilitate participation in task-oriented training, have been used successfully. In recent years, case reports of assistive technologies in acute stroke have been emerging (12–14). Even though in our resent research team's study, the use of orthosis was highly satisfying to participants, no additional benefit was found in wearing a dynamic hand orthosis during unilateral task-oriented training in the early subacute period (15).Furthermore, strong imaging technological evidence on the use of dynamic hand orthoses combined with task-oriented training in subacute stroke remains scarce (9).

Functional near-infrared spectroscopy (fNIRS) can be used to monitor the direct and indirect brain regions involved in complex motor learning and is not limited by dynamic posture (16, 17). Persons with stroke can complete fNIRS examination not only in static states such as sitting and standing but also in dynamic statuses, such as playing an instrument or grasping items. fNIRS has many advantages, including non-invasiveness, low cost, portability, and ease of operation (18, 19), and is not affected by metal in the body, such as blood vessel stents or spring coils, which are forbidden when scanned by functional magnetic resonance imaging (fMRI). This makes it favorable for studying the cortical response to simple and complex motor stimulation in stroke patients. Using fNIRS, research outcome measures, such as oxyhemoglobin concentrations (HbO), deoxyhemoglobin concentrations (dex-HbO), or beta value, derived from these data could be used to describe the activation intensity, and the number of activation channels could be used to describe the scope of individual activation (20).

Region of interest (ROI), defined as cortical regions functionally connected and cooperating to achieve skilled motor or other performance (16), such as motor-related primary motor cortex (M1), sensorimotor cortex (SMC), supplementary motor cortex, pre-supplementary motor area (Pre-SMA), premotor cortex (PMC), and prefrontal cortex (PFC), can reflect cerebral cortical reorganization during the process of stroke recovery. And by adding various tasks, it can be used to evaluate the changes in the ROI after rehabilitation training (21–24). For complex motor stimulation, ROI research focuses mainly on the primary motor area (M1), sensorimotor cortex, premotor cortex, and prefrontal cortex. In an fNIRS study, the participant wears a head cap embedded with light sources and detectors positioned over the ROI of the brain under investigation, and brain activity *via* statistical analysis of the evoked changes in oxyhemoglobin and deoxyhemoglobin concentration are observed.

Therefore, we explored, using a grip-release ring motor task, the activated changes of ROI utilizing fNIRS technology on persons with stroke who received unilateral task-oriented therapy with a hand orthosis in the early subacute stroke period (7 days−3 months). The study aimed to find a sensitive motor task and region of interest to evaluate the effectiveness and mechanism of this rehabilitation method by fNIRS technology for the next randomized controlled trials (RCTs).

## Case presentation and methods

### Participants

The study was approved by the Regional Committee for Medical and Health Research Ethics (No.2017/1915 REK sør-øst D) and the China Rehabilitation Research Center Ethics Committee (No.CRRC-IEC-RF-SC-005-01). Inclusion criteria were: ① First Stroke, ② Right hemiplegia and right-handed, ③ Age > 18 years, ④ 14–90 days since stroke (±3 days), ⑤ Partial finger movement (defined as ≥ 10 degrees of active finger flexion), and ⑥ Provided written informed consent.

Exclusion criteria were: ① Full finger extension; ② Language and/or cognitive impairments that preclude the person from following instructions (defined as Mini-Mental State Examination, MMSE ≤ 20 score); ③ and Other health conditions that preclude the person from undergoing rehabilitation, such as severe depression, anxiety, mental symptoms, or internal disease. A total of eight right-handed and right hemiplegia participants were recruited from the neurorehabilitation ward in the China Rehabilitation Research Center (CRRC).

**Figure 1:**
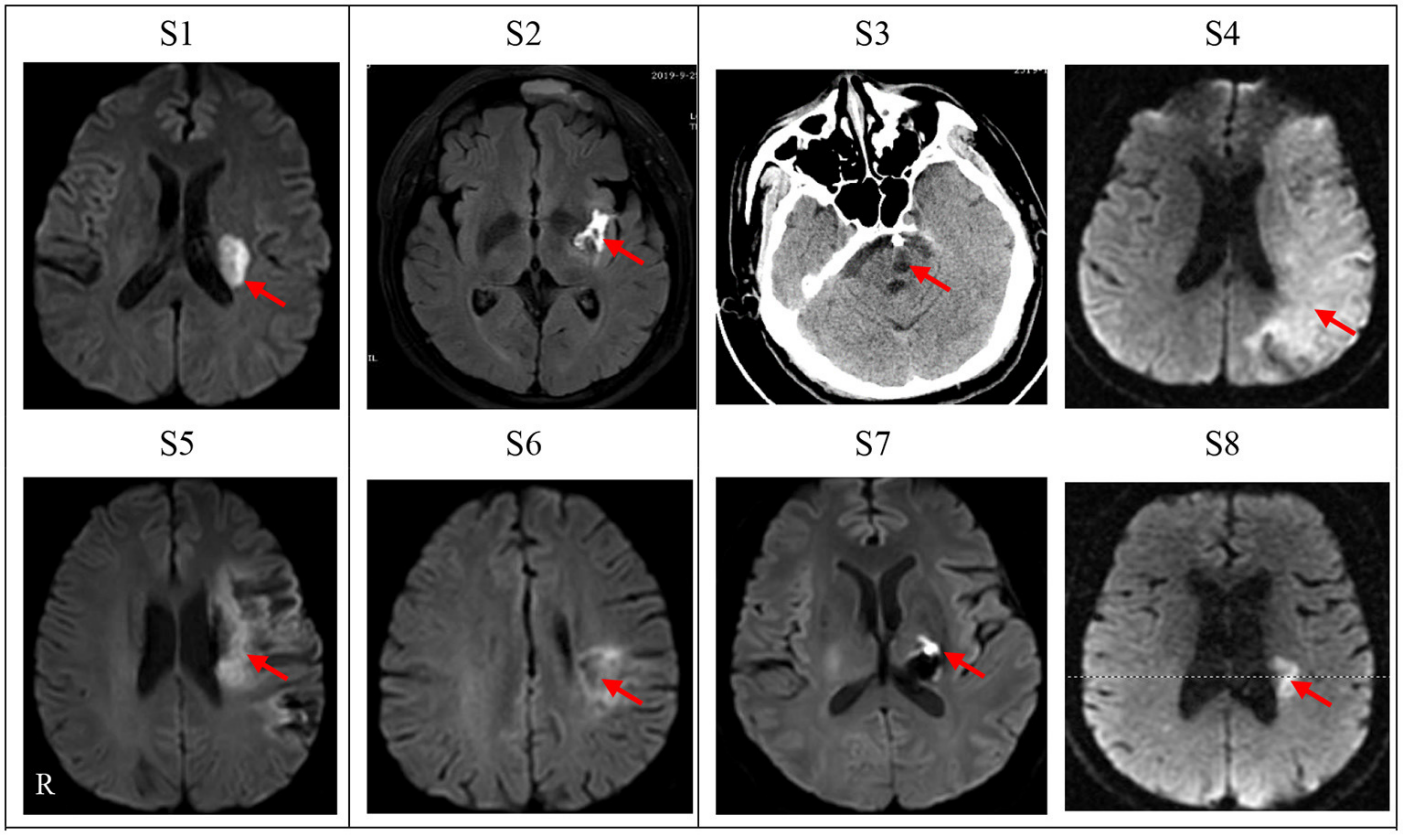
Diffusion weighted-MRI Images or CT images for cases *n* = 8). Lesion location of each subject was shown as diffusion weighted-MRI scan images except one subject (S3) with CT scan who had an intracranial metal clip. S1–S8 denotes the eight patients in this study. Left side of figures represents right side of the subjects. R, Right.

These participants were between the ages of 47 years and 72 years (58.88 ± 9.19); two individuals were female and six individuals were male, while six suffered ischemic stroke and two hemorrhagic stroke. The total amount of therapy ranged from 3 to 4 h (3.25 ± 0.38) per day. The total amount of medication ranged from 4 to 14 (10.88 ± 3.09).The time from stroke to admission ranged from 35 to 92 days (54.50 ± 17.86). A full outline of the general characteristics of persons with stroke can be found in Table 1. Diffusion weighted-MRI Images or CT images are shown for each participant in Figure 1. The white or black areas indicated by the red arrow were the ischemic or hemorrhagic regions in MRI or CT images.

**Table 1:** General characteristics of persons with stroke (*n* = 8).

**Subjects**	**Age (years)**	**Sex^a^**	**Time from stroke to admission(days)**	**Lesion location**	**Stroke type**	**NIHSS**	**Total amount of therapy (hour)**	**Medications (n)**
S1	47	M	50.00	L Basal ganglia, periventricular	CI	3	3	10
S2	53	M	35.00	L Basal ganglia	IH	4	3	10
S3	68	F	44.00	L Pons	CI	5	4	14
S4	55	M	50.00	L frontal, parietal, temporal	CI	5	3	12
S5	62	M	44.00	L frontal, temporal,	CI	3	3	12
S6	65	F	92.00	L frontal, parietal, temporal,	CI	7	3.5	12
S7	49	M	53.00	L Thalamus	IH	7	3.5	4
S8	72	M	68.00	L Basal ganglia, periventricular	CI	4	3	13
Mean ± SD	58.88 ± 9.19	-	54.50 ± 17.86	-	-	4.31 ± 0.79	3.25 ± 0.38	10.88 ± 3.09

a *Sex; F, Female; M, Male; L, Left-side; R, Right-side; IH, Intracerebral Hemorrhage; CI, Cerebral Infarct*.

### Intervention

All persons with stroke received unilateral task-oriented therapy for the affected upper extremity, 5 times/week for 4 consecutive weeks. This included 10 min gross motor training, 10 min fine motor training, and 10 min intensive training. Each time they wore a hand orthosis device (SaeboGlove) on the affected hand for 30 min and another 30 min without the orthosis. All persons with stroke were under the supervision of a licensed OT therapist during the training. Training was undertaken either while the participant was standing or sitting.

### Experimental setup

#### fNIRS outcome measurements

##### Experimental design

fNIRS measurements were performed on the same day of recruitment and the day after 4 weeks of intervention. The subject was instructed to grip-release a 20-db (9.06-kg) grip ring with the hemiplegic hand. The experiment used a block design with five repeated cycles of 30 s rest and 15 s movement. The experiment started with a 10 s pre-scan and a 30 s rest time, which were not used for statistical analysis. The participant was instructed to repeatedly grip-release with the hemiplegic hand five times at a steady speed, guided by an auditory metronome during the 15 s period. The task required the participant to relax the non-grasping hand and to avoid any movements other than those required for the motor tasks during the performance of the one-handed grip-release task. Activation and relaxation were monitored and recorded by the tester.

##### Data acquisition

**Figure 2:**
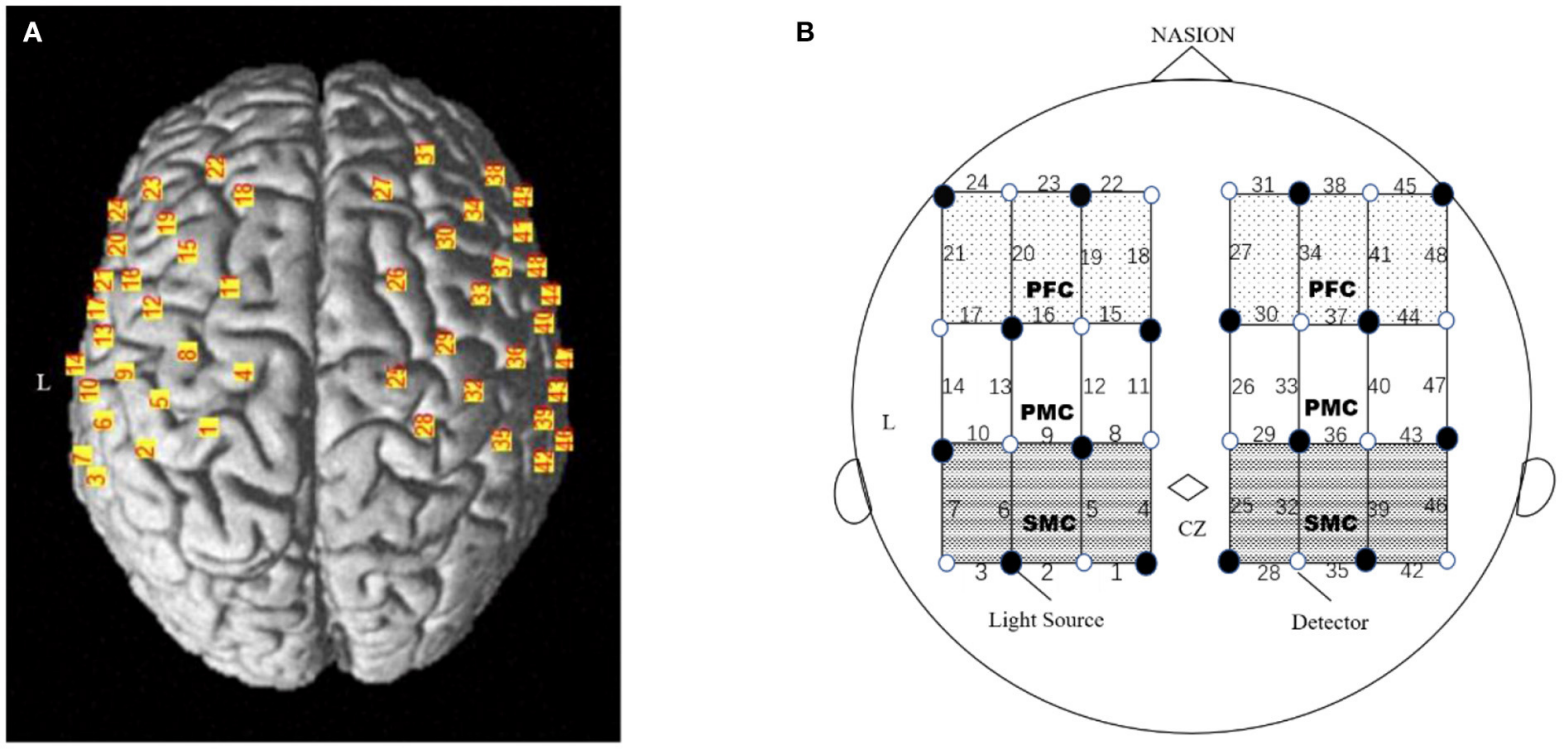
Experimental setting of the optodes. **(A)** 3D location of the optodes exposed to the brain surface of a standard brain. A total of 32 optodes. **(B)** The channels covering the SMC, PMC and PFC, comprising 8 light source fibers (black dot) and 8 detectors (white dot), were arranged on the scalp to enable 48 channel measurements. The Cz position of the international 10–20 system was the marker for ensuring the replicable placement of the optodes.

The optical signal was measured using a 48-channel near-infrared spectroscopy (NIRS) machine (Hitachi, ETG-4100). Near-infrared lights with wavelengths of 695 nm and 830 nm were guided by optical fiber bundles and transmitted into the brain through the cranium, The fNIRS raw light signals were converted to a measure of the change in optical absorption over time for each pair to measure changes in the oxyhemoglobin and deoxyhemoglobin concentration at a sampling frequency of 10 Hz during the motor task. Two plastic probe holders (4 × 4 matrices) with 24 channels positioned on either side of the head were placed on the scalp over the patient's bilateral motor-related areas. A total of eight sources and eight detector fiber bundles were positioned on each plastic probe holder. The probes were placed 3 cm away from each other, to monitor the cortical activation over two 9 × 9 cm rectangular fields of view (25).The participants' head movements were strictly suppressed during the whole task using a chinrest, and the light probes were fixed through an integral plastic sheet to deal with motion artifacts. The electroencephalography (EEG) International 10–20 system Cz, C3, C4, F3, and F4 anatomical measurements were used as reference points to ensure that the optical probe setup was placed over six ROIs (26), including the bilateral sensorimotor cortex (SMC), premotor cortex (PMC), and prefrontal cortex (PFC). A three-dimensional (3D)-digitizer was used to record the exact locations of each fNIRS probe for a standard brain before converting these coordinates into the locations of the 48 channels in an estimated Montreal Neurological Institute (MNI) space using the MATLAB toolbox NIRS-SPM (27). The positioning of the 48 channels on a reconstructed 3D brain (23) is shown in Figure 2A. Based on the mean MNI coordinates and Brodmann's area (BA) correspondences, the six ROIs were covered by the following channels for the left and right hemispheres: the left SMC was covered by channels 4, 5, 6, 8, and 9 (both sides of C3); the right SMC was covered by channels 25, 29, 32, 36, and 39 (both sides of C4); the left PMC was covered by channels 11, 12, 13, 15, and 16; the right PMC was covered by channels 26, 30, 33, 37, and 40; the left PFC was covered by channels 18, 19, 20, 22, and 23; and the right PFC was covered by channels 27, 31, 34, 38, and 41. Based on the modified Beer-Lambert law, values of oxyhemoglobin were acquired following changes in levels of cortical concentration. HbO level was selected since it is reliable and sensitive to changes in cerebral blood Flow (28).The source-detector probe geometry of the fNIRS system is shown in Figure 2B.

##### Processing and image analysis 1

To estimate the effect on cortical activation during the motor task, we performed channel-based analysis. As a first-level analysis, we made individual intrasubject contrast images comparing baseline rest periods and motor periods; the general linear regression model (GLM) analysis with hemodynamic response curve to model the oxyhemoglobin response during stimulus condition was performed to calculate the beta value as the individual task-related activity (20). Any data with obvious motion artifacts or damaged channels in any block/rest period were automatically and manually excluded from further analysis. Based on the beta value, topography was plotted on the location of the channels. Next, a 0.01–0.1 Hz bandpass filter was applied to remove global fluctuation due to heartbeat (0.8–2.0 Hz), respiration (0.1–0.33 Hz), and Mayer waves (0.1 Hz or lower) (29, 30); Gaussian smoothing was applied to estimate and remove temporal autocorrelation. The wavelet-minimum description length (MDL)-based detrending algorithm was used to correct any signal distortion.

##### Processing and image analysis 2

To describe oxyhemoglobin concentration time course for all the subjects, Software MATLAB toolbox HOMER was used to process the original data. For each subject, 5 s of data before the start of each task to 35 s of data after the start were selected for analysis. Baseline “0” value was defined as the time when the motor task started; a selected 5 s of data before “0” value was used as the baseline, and baseline correction was taken each time when extracting the data. After extracting the data related to the task and performing baseline correction, task-related oxygenated hemoglobin concentration changes of the subjects following time change on the channels of ROIs were obtained by a superposed average of each block. Time course maps on six ROIs of each subject and group were performed.

#### Clinical outcome measures

Clinical outcome measures included the Grip Strength Evaluation (Jamar Digital Hand Dynamometer, kg), the Action Research Arm Test (ARAT) (31), and the Fugl-Meyer Assessment Upper Extremity Scale (FMA-UE, or FMA-arm) (32). Each participant was initially evaluated at the time of recruitment and 4 weeks after unilateral task-oriented training with an orthosis.

### Data analysis

All statistical analysis were performed using SPSS 19.0. The parameters used for the data analysis were FMA-arm, ARAT, and Grip strength of upper extremity function. Measurement data conforming to normal distributions are presented as the mean ± standard deviation (mean ± SD); paired *t*-tests were used to identify the training effects before and after the intervention. Paired *t*-test was used to compare beta values of six ROIs before and after the intervention. Significance was set at *P* < 0.05. The confidence interval was 95%.

## Outcome measures results

### fNIRS outcomes

#### Individual data outcomes of activation intensity

**Figure 3:**
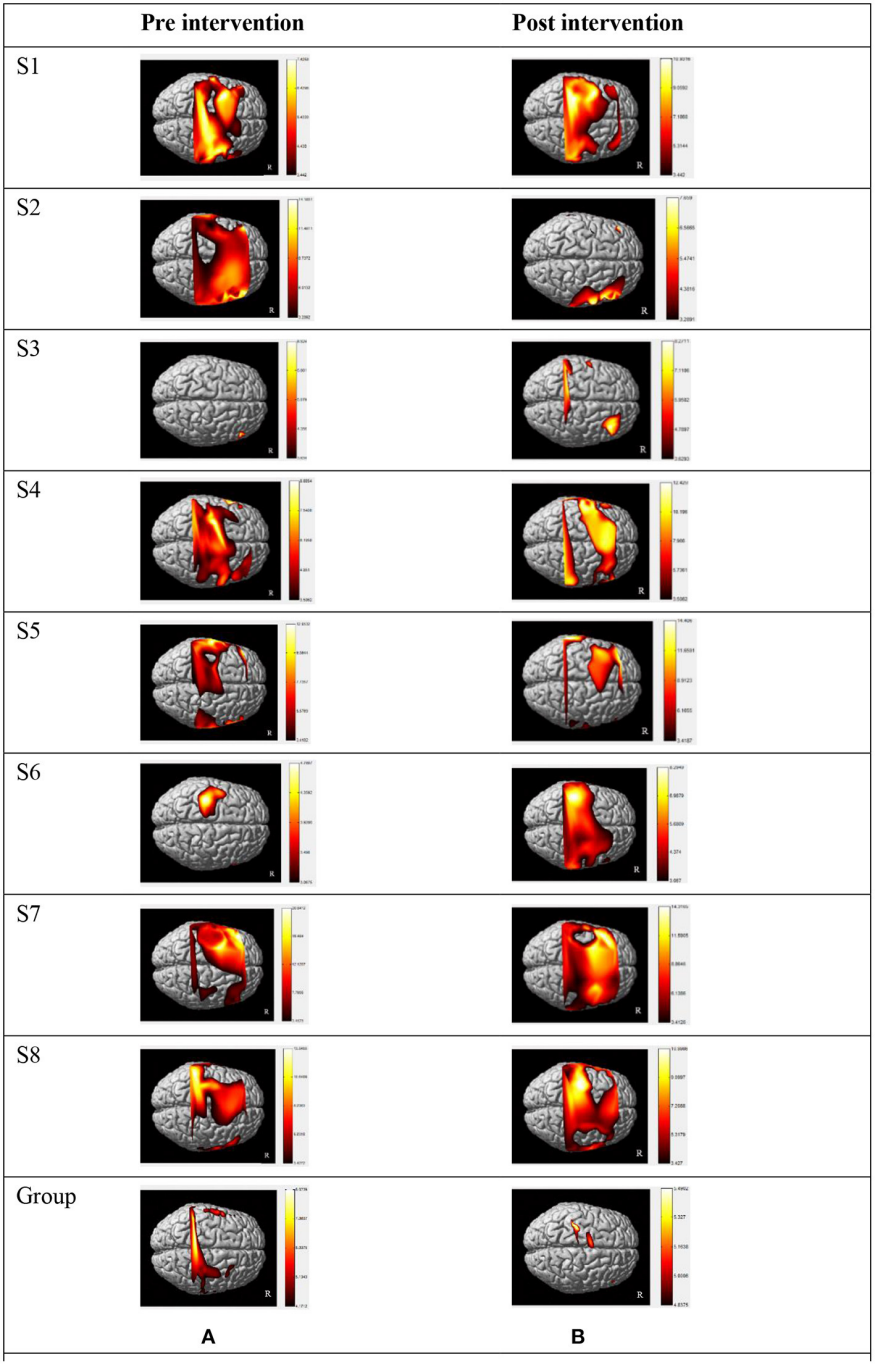
Individual activation maps and group activation maps of HbO during a motor task generated using the NIRS-SPM pre **(A)** and post **(B)** intervention. Patients exhibited high activity levels (yellow color) in different brain areas. **(A)** Individual and group activation dorsal view map of HbO during grip-release task in pre intervention generated using the NIRS-SPM (*p* < 0.05). **(B)** Individual and group activation dorsal view map of HbO during grip-release task in post intervention generated using the NIRS-SPM (*p* < 0.05). S1–S8: the eight patients in this study. Right side of figures represents right side of the subjects. Group: all participant's group activation dorsal view map of HbO in pre and post R: Right.

There was a discernible trend of beta value derived from oxyhemoglobin concentrations by fNIRS: from pre- intervention to post- intervention, S1, S2, S3, S6, S7, and S8 (*n* = 6,75.0%) showed an obvious trend for decreased mean beta value in LSMC (contralateral SMC), and the same increased or decreased trend ratio (*n* = 4,50.0%)in RSMC (ipsilateral SMC). S1, S2, S5, S6, and S7 (*n* = 5,62.5%) showed a trend for increased mean beta value in LPMC (contralateral PMC), whereas S1, S2, S4, S6, S7, and S8 (*n*= 6,75.0%) showed a trend for decreased mean beta value in RPMC (ipsilateral PMC).S1, S2, S4, S5, S6, and S8 (*n* = 6,75.0%) showed a trend for increased mean beta value in LPFC (contralateral PFC), whereas S1, S2, S3, S4, S6, and S7 (*n*= 6,75.0%) showed a trend for decreased mean beta value in RPFC (ipsilateral PFC).All these data indicated that, accompanied by the recovery of stroke, both cerebral hemispheres were activated when persons with right hemiplegia were completing an upper limb movement. The activation intensity of LSMC, RPMC, and RPFC decreased while activation intensity of LPMC and LPFC increased after the intervention. Mean beta values of six ROIs pre and post intervention of all the subjects are shown in Table 2. Before intervention, S1, S2, S4, S5, S7, and S8 showed the maximum mean beta value of six ROIs located in LSMC. S3 showed the maximum mean beta value of six ROIs located in RSMC, while S6 showed the maximum mean beta value of six ROIs located in LPMC. After intervention, S1, S2, S6, S7, and S8 showed the maximum mean beta value of six ROIs located in LPMC, while S3 showed the maximum mean beta value of six ROIs located in RPMC, and S4 and S5 showed the maximum mean beta value of six ROIs located in LSMC. The changes from pre-intervention to post-intervention according to β value are shown in Table 3.The fNIRS activation maps for the task relative to the resting baseline before and after the intervention are shown in Figure 3.

**Table 2:** Mean beta value (mol)of 6 ROIs pre and post intervention of all the subjects.

**Subjects**	**RSMC**		**LSMC**		**RPMC**		**LPMC**		**RPFC**		**LPFC**	
	**Pre**	**Post**	**Pre**	**Post**	**Pre**	**Post**	**Pre**	**Post**	**Pre**	**Post**	**Pre**	**Post**
S1	4.97	0.78	5.61^*^	0.29	2.63	2.46	5.37	14.0^**^	5.08	4.48	3.54	5.15
S2	3.36	2.09	8.33^*^	−6.97	1.29	−1.10	7.27	23.6^**^	2.61	0.95	7.49	8.52
S3	0.71^*^	0.73	−0.39	−3.69	0.41	1.23^**^	−1.45	−3.07	0.20	−0.07	−1.60	−3.92
S4	0.541	1.33	1.69^*^	3.64^**^	0.99	0.95	0.56	0.12	0.47	−0.80	−0.12	0.96
S5	0.29	2.46	1.93^*^	4.15^**^	0.49	2.16	0.91	1.81	0.18	1.38	−0.01	0.73
S6	5.55	0.26	6.23	4.82	6.27	2.46	8.10^*^	9.70^**^	7.56	−0.36	6.87	7.43
S7	2.75	0.80	4.11^*^	1.03	3.23	0.85	3.27	4.78^**^	2.04	1.47	2.98	2.33
S8	1.76	3.18	5.00^*^	4.22	2.75	2.18	4.41	2.68	2.05	2.13	4.35	4.51^**^
Mean	2.49	1.45	4.06^*^	0.94	2.26	1.40	3.55	6.70^**^	2.52	1.15	2.94	3.21

*Pre, pre-intervention; Post, post-intervention; L(R)SMC, Left (Right)sensorimotor cortex; L(R)PMC, Left (Right)premotor cortex; L(R)PFC, Left (Right) prefrontal cortex*.

* *the maximum mean beta value of 6 ROIs pre intervention*.

** *the maximum mean beta value of 6 ROIs post intervention*.

**Table 3:** Change Patterns of brain regions from pre-intervention to post-intervention according to β value.

**Brain region**	**Patterns of change**	
	**Decreased**	**Increased**
	**(Subject number)**	**(Subject number)**
**A. Lesioned hemisphere, Contralateral, left**		
1.LSMC	75.0% (S1 S2 S3 S6 S7 S8)	25.0% (S4 S5)
2.LPMC	37.5% (S3 S4 S8)	62.5% (S1 S2 S5 S6 S7)
3.LPFC	25.0% (S3 S7)	75.0% (S1 S2 S4 S5 S6 S8)
**B. Non-Lesioned hemisphere, Ipsilateral, right**		
1.Right SMC	50.0% (S1 S2 S6 S7)	50.0% (S3 S4 S5 S8)
2.Right PMC	75.0% S1 S2 S4 S6 S7 S8)	25.0% (S3 S5)
3.Right PFC	75.0% (S1 S2 S3 S4 S6 S7)	25.0% (S5 S8)

*S, Subject; L(R)SMC, Left (Right) sensorimotor cortex, L(R)PMC, Left (Right) premotor cortex; L(R)PFC, Left (Right) prefrontal cortex*.

#### Group data outcomes of activation intensity

Single-sample *t*-tests (test value β = 0) were performed on all subjects before and after the intervention by using beta values estimated from oxyhemoglobin data. Significant activation channels were defined as channels with *P* < 0.05 beta values during a grasp-release ring task compared to baseline (beta value = 0). Before the intervention, a total of 24 channels were activated; the task showed significantly increased activation (*P* < 0.05) on the LSMC in channels 4, 6, and 9 and on the RSMC in channels 29, 32, 36, and 39. There was also increased activation on the LPMC in channels 11, 12, and 13 and on the RPMC in channels 26, 30, and 33. No significant activations were observed on the LPFC in all the channels, but the task showed significantly increased activation on the RPFC in channels 27, 31, and 34. After the intervention, a total of 22 channels were activated; the task showed significant activation on the RSMC in channels 29, 32, 36, and 39, but no significant activations were observed on the LSMC in all the channels. The task also showed increased significant activation on the LPMC in channels 12, 13, and 16 and on the RPMC in channels 26, 33, and 40. The task showed increased significant activation on the LPFC in channels 20 and 23 and on the RPFC in channel 38.

The number of significantly activated channels in the bilateral hemisphere before the intervention was right hemisphere (*n* = 10)>left hemisphere (*n* = 6); after the intervention it was right hemisphere (*n* = 8)>left hemisphere (*n* = 5).This result suggested that right hemisphere activation plays a dominant role, while bilateral hemispheres' activation decreased after training.

The number of significantly activated channels in ROI before the intervention was RSMC (*n* = 4)>LSMC = LPMC = RPMC = RPFC (*n* = 3)>LPFC (*n* = 0); after the intervention it was RSMC (*n* = 4)>LPMC = RPMC (*n* = 3)>LPFC (*n* = 2)>RPFC (*n* = 1)>LSMC (*n* = 0).This result suggested that RSMC activation is a dominant brain area, while LSMC and RPFC activation decreased and LPFC increased after training. Activated channels for all the persons with stroke are shown in Table 4.

**Table 4:** Activated channels pre and post intervention of all the subjects in group analysis.

	**Activated channel**	**n**	**LSMC**		**RSMC**		**LPMC**		**RPMC**		**LPFC**		**RPFC**	
			**Cha**	P^*^	**Cha**	P^*^	**Cha**	P^*^	**Cha**	P^*^	**Cha**	P^*^	**Cha**	P^*^
Pre	1 2 3 4 6 7 9 10 11 12 13 26 27 28 29 30 31 32 33 34 35 36 39 44	24	4	0.021	29	0.005	11	0.043	26	0.007	-		27	0.011
			6	0.007	32	0.005	12	0.015	30	0.001			31	0.036
			9	0.032	36	0.023	13	0.034	33	0.012			34	0.025
					39	0.033								
Post	2 3 7 12 13 16 17 20 23 26 28 29 32 33 36 38 39 40 42 43 46 47	22	-	29	0.022	12	0.044	26	0.022	20	0.181	38	0.014	
				32	0.002	13	0.019	33	0.016	23	0.02			
				36	0.007	16	0.01	40	0.003					
				39	0.02									

*Pre, pre-intervention; Post, post-intervention; cha, channel; L(R)SMC, Left (Right) sensorimotor cortex; L(R)PMC, Left (Right)premotor cortex; L(R)PFC; Left (Right) prefrontal cortex*.

* *P <0.05*.

#### Time course

**Figure 4:**
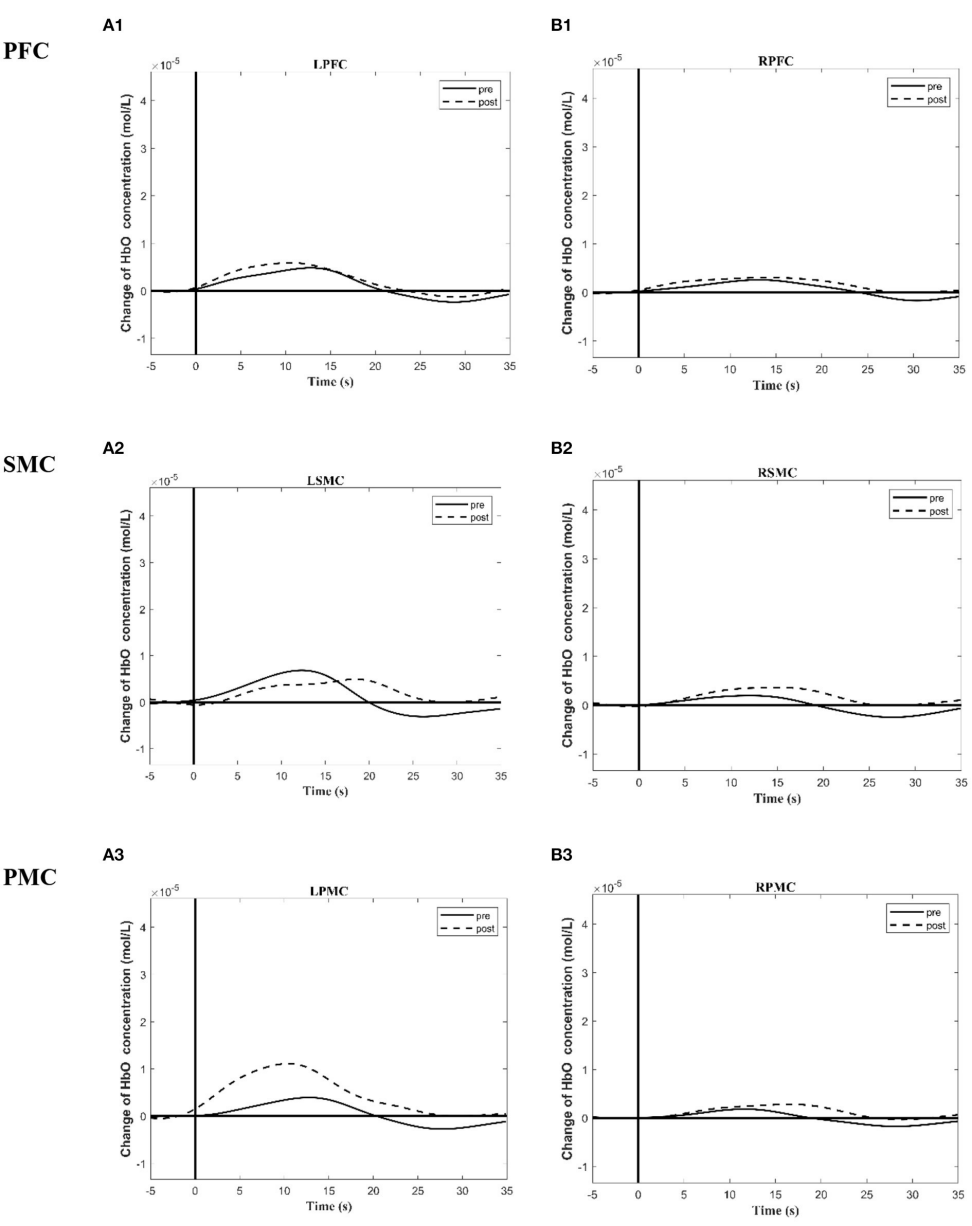
Group time course maps of HbO concentration change during a motor task generated using the MATLAB HOMER toolbox pre and post the intervention. **(A1–A3)** Group activation time course maps of HbO concentration change on LPFC **(A1)**, LSMC **(A2)**, LPMC **(A3)** during grip-release task pre and post the intervention. **(B1–B3)** Group activation time course maps of HbO concentration change on RPFC **(B1)**, RSMC **(B2)**, and RPMC **(B3)** during grip-release task pre and post the intervention. The y-axis displays concentration changes of hemoglobin in moles per liter. The change of HbO concentration is indicated by solid line (pre intervention) and dotted line (post intervention). L (R)SMC, Left (Right)sensorimotor cortex; L (R)PMC, Left (Right)premotor cortex; Left (Right)PFC, Left (Right)prefrontal cortex.

Tables 5, 6 summarized the results of the average individual peak, group Peak (maximum HbO concentration, mol/l), and Latency (time to reach peak, s)of all eight subjects from their baseline by fNIRS over six ROIs during a 45 s grip-release ring task. The peak of LSMC, LPMC, and LPFC was greater than that of contralateral ROIs pre and post intervention, with the highest peak in LSMC pre intervention and LPMC post intervention. The latency of LSMC, LPMC, and LPFC was shorter than that of contralateral ROIs pre intervention, with the shortest latency of LSMC pre intervention. And post intervention, the latency of LPMC and LPFC was shorter than that of contralateral ROI, with the smallest latency of LPFC. Change of HbO concentration as measured by NIRS over six ROIs in group during motor task was observed in Figure 4; high resolution figures can be seen in Supplementary materials. The task-related response patterns consist of a rapid increase in HbO concentration which later decreased to baseline.

**Table 5:** The average individual and group Peak (mol/l) of all the subjects on 6 ROI pre and post intervention during grip-release ring task.

**Subjects**	**RSMC**		**LSMC**		**RPMC**		**LPMC**		**RPFC**		**LPFC**	
	**Pre**	**Post**	**Pre**	**Post**	**Pre**	**Post**	**Pre**	**Post**	**Pre**	**Post**	**Pre**	**Post**
S1	3.96E-06	3.56E-06	4.89E-06	1.09E-05	3.41E-06	1.28E-06	6.50E-06	5.25E-06	2.46E-06	2.46E-06	3.48E-06	4.29E-06
S2	5.39E-06	5.72E-06	5.75E-06	1.73E-06	6.26E-06	5.16E-06	4.64E-06	−8.02E-07	5.44E-06	4.70E-06	6.07E-06	−7.09E-07
S3	4.72E-07	−5.50E-07	1.88E-06	5.53E-06	1.14E-06	2.25E-06	1.91E-06	1.05E-05	2.70E-06	5.72E-06	1.42E-06	6.26E-06
S4	3.12E-06	2.71E-06	6.89E-06	6.44E-06	2.61E-06	3.05E-06	4.91E-06	2.15E-05	6.87E-06	5.76E-06	1.33E-06	8.64E-06
S5	4.7E-06	5.35E-06	1.06E-05	3.39E-06	1.5E-06	2.94E-06	6.03E-06	4.61E-05	−1.30E-07	3.93E-06	6.08E-06	1.88E-05
S6	1.37E-06	4.63E-06	3.1E-06	5.56E-06	1.81E-06	3.35E-06	1.69E-06	3.40E-06	1.82E-06	2.26E-06	1.94E-06	1.94E-06
S7	1.01E-06	4.23E-06	2.11E-05	9.70E-06	1.39E-06	5.87E-06	1.32E-05	9.93E-06	2.12E-06	3.45E-06	2.29E-05	9.49E-06
S8	1.07E-06	5.69E-06	6.41E-06	1.15E-05	1.32E-06	4.33E-06	−4.37E-07	2.82E-06	2.13E-06	2.45E-06	9.28E-07	8.70E-06
Group	2.64E-06	3.92E-06	7.58E-06	6.84E-06	2.43E-06	3.53E-06	4.81E-06	1.23E-05	2.93E-06	3.84E-06	5.52E-06	7.17E-06

*Pre, pre-intervention; Post, post-intervention; L(R)SMC, Left (Right)sensorimotor cortex; L(R)PMC, Left (Right)premotor cortex; L(R)PFC, Left (Right) prefrontal cortex*.

**Table 6:** The average individual and group Latency (s) of all the subjects on 6 ROI pre and post intervention during grip-release ring task.

**Subjects**	**RSMC**		**LSMC**		**RPMC**		**LPMC**		**RPFC**		**LPFC**	
	**Pre**	**Post**	**Pre**	**Post**	**Pre**	**Post**	**Pre**	**Post**	**Pre**	**Post**	**Post**	**Pre**
S1	17.4	11.6	16.2	13.4	16.3	16.8	14.2	12.3	16.6	15.8	15	15.1
S2	13	14.6	8.8	14	12.9	14.6	13.5	8.9	12.9	15.2	11.4	5
S3	33.5	34.9	12.4	32.4	25.6	34	14.6	29	11.9	6	9.7	9.3
S4	8.9	17.1	10.2	20.5	8.7	15.7	9.5	12.2	15.9	7	9.2	14.4
S5	11.9	7	8.9	21	13.2	21.5	9.4	7.3	13.2	21.8	8.7	6.9
S6	34.9	14.3	13	13.4	34.9	13.8	34.9	12.9	34.9	12.9	34.7	12.2
S7	21.5	18.1	12.7	11.9	20.6	18	12.8	12.1	20.4	18	13.7	13.7
S8	9.7	10.6	15.8	15.2	8.8	17.8	20.3	19.7	14.1	11.8	16.3	14.3
Group	18.85	16.025	12.25	17.725	17.625	19.025	16.15	14.3	17.488	13.562	14.838	11.363

*Pre, pre-intervention; Post, post-intervention; L(R)SMC, Left (Right)sensorimotor cortex; L(R)PMC, Left (Right)premotor cortex; L(R)PFC, Left (Right) prefrontal cortex*.

### Clinical outcomes

All the subjects had an improvement after intervention in three clinical tests. In group data, FMA-arm score ranged from 15 to 49 at pre-test and ranged from 22 to 59 at post-test. The mean ± SD FMA-arm score for all the subjects at pre-test was 34.25 ± 11.39, compared with 46.50 ± 11.74 at post-test, which reflected a significant improvement in upper limb and hand gross motor function in group analysis (*P* = 0.024^*^). The ARAT score ranged from 10 to 35 at pre-test and ranged from 10 to 48 at post-test. The mean ± SD ARAT score for all the subjects at pre-test was 20.00 ± 10.17, compared with 35.38 ± 15.58 at post-test, which reflected a significant improvement in fine motor function of hand and finger grasp-release function in group analysis (*P* = 0.006^*^).Grip strength score ranged from 1 to 8.2 at pre-test and ranged from 2 to 14 at post-test. The mean ± SD Grip strength score for all the subjects at pre-test was 5.14 ± 4.25 kilograms, compared with 6.50 ± 4.60 kilograms at post-test, which reflected a significant improvement in grip strength function in group analysis (*P* = 0.007^*^). FMA-arm score, ARAT score, and Grip strength score of all the persons with stroke were provided in Table 7.

**Table 7:** FMA-arm score, ARAT score and Grip strength score of all the subjects (*n* = 8).

	**Subjects**	**1**	**2**	**3**	**4**	**5**	**6**	**7**	**8**	**Mean ±SD**	**P**
FMA-arm	Pre	35	40	30	49	44	22	15	39	34.25 ± 11.39	0.024^*^
	Post	42	51	46	59	56	22	42	54	46.50 ± 11.74	
ARAT	Pre	10	21	35	31	12	10	13	28	20.00 ± 10.17	0.006^*^
	Post	22	47	57	40	33	10	26	48	35.38 ± 15.58	
Grip strength (Kilogram)	Pre	3.9	1	8.2	13	4	2	1	8	5.14 ± 4.25	0.007^*^
	Post	4	7	8	14	3	2	2	12	6.50 ± 4.60	

*FMA-arm, Fugl-Meyer Assessment of the arm; ARAT, Action Research Arm Test; Pre, Pre-intervention; Post, Post- intervention*,

* *P <0.05*.

## Discussion and conclusion

### fNIRS

fNIRS can be used to monitor changes in cortical hemodynamics with motor skill learning. The study of Ikegami and Taga showed that fNIRS could be used to detect the changes of the contralateral sensorimotor cortex in upper limb tasks, and could reflect the corresponding relationship between the cerebral cortex and the progress of motor learning (33).Previous fMRI research shows that, in healthy right-handed people, completing a motor task with the right hand is accompanied by dominated activation on the contralateral hemisphere, while the activation on the ipsilateral hemisphere is relatively less (34). But in people with stroke, studies have demonstrated that functions, previously the responsibility of damaged areas of the cortex, may be taken over by adjacent areas or by areas in the contralateral hemisphere (11). This means the ipsilateral brain network of the paralyzed hand was recruited and the activity of the paralyzed side is accompanied by the activation of bilateral motor cortex. With the stroke recovery, the activation model tended to the dominant contralateral activation as the healthy person. The change of the total number of significant activation channels suggests that the bilateral activation scope of the task-related regions decreased, which means when participants completed the same task after rehabilitation training, the need for recruitment of bilateral hemispheres was reduced. Meanwhile, more right hemisphere (undamaged hemisphere) activation was accompanied by 4 weeks of intervention during subacute stroke period. This result was consistent with other previous mechanic studies in hand motor function (35–38).

Recent research on complex motor stimulation tasks, such as the task of using chopsticks, shows that relevant ROI involved M1, SMC, and PMC (39). Bilateral SMC activation is related to motor initiation and regulation. In addition, the PFC is responsible for higher-level information processing, such as motor judgment, planning, and error correction. In SMC, our result was consistent with most studies that reported a recovery of motor function was accompanied by a shift in SMC activation from the unaffected hemisphere to the affected hemisphere (24, 35, 39, 40). Enhanced activation of the unaffected SMC may represent the mechanism of recovery for the paralyzed upper extremity. In recent years, Tamashiro found that patients with brain activity that transferred to the unaffected (contralateral) cerebral cortex showed better motor recovery than those with no activity transfer. Therefore, the evaluation activation changes may help guide therapeutic interventions and the evaluation of prognosis (40, 41).

In PMC, there were two opposite theories about the change of PMC area after stroke. The first theory stated that motor function recovery was supported by the sensory motor network of the damaged hemisphere, including contralateral, medial PMC, extral pre motor, and primary motor (MI) (42, 43). The second theory stated that uninjured ipsilateral dorsolateral PMC was related to motor recovery (44–46).The results of this study are consistent with the first theory. In PFC, some studies suggest that the neural plasticity of the cerebral cortex is accompanied by skill learning. Leff's study showed a short motor skill learning process could lead to changes in prefrontal activation (47). However, not all new tasks can activate PFC, possibly because some motor skills are very complex and independent of executive control during the initial stage of learning. In our study, by executing a grip ring task, bilateral prefrontal regions can be activated in subacute stroke recovery. The activation intensity of ipsilateral (LPFC)was increased after intervention. It suggested that the improvement of motor function is related to the intervention.

Some data demonstrate that oxyhemoglobin and deoxyhemoglobin returned to the baseline 9–10 s after motor stimulation. But because fNIRS relies on hemodynamic responses, which take some time to occur, oxyhemoglobin and deoxyhemoglobin signals have a lag of roughly 4 s. Motor stimulation with a duration of 10–15 s can achieve satisfactory results (48). Therefore, the current RCT design mostly adopts a stimulation time of 10–15 s. However, different from the motor mode of healthy people, the sports endurance of patients with subacute stroke is reduced, and muscle fatigue occurs more easily; in addition, some patients have attention disorders. Considering these factors that may affect the adherence of fNIRS tests, the design adopts the repeated trial of five blocks to ensure completion. In this case series, changes in the range and intensity of brain activation during stroke recovery were shown in the results. Despite different types of motor stimulation, fNIRS instrumentation, and stimulation protocols, similar temporal patterns of hemoglobin concentration change have been observed. Broadly, this response consists of a rapid increase in HbO which then decreased to baseline, and the most reliable cortical hemodynamic response is 2–20 s following a motor stimulus (48). This pattern of change typifies the hemodynamic response to brain activation. Our time course outcomes are consistent with prior research results. However, as patients were in the early subacute phase after stroke, their improvement might have (partly) been due to several factors: spontaneous recovery, the use of unilateral task-oriented training combined with dynamic orthosis, other medicines or training, or a combination of these factors. Our results could not distinguish which factor is dominant.

In this study, although bilateral activation existed in the recovery process, which was consistent with previous studies (49, 50), the intensity of activation varies in different brain regions (43, 51). Further RCT study should compare differences in activation intensity in bilateral ROI between groups. If a paired sample design is assumed to compare changes of β value within groups in ipsilateral SMC, to achieve 80% power of a test, at alpha = 0.05, 1-β = 0.8, δ = 3.124,a sample size of 505(two-tailed)in each group will be sufficient. These results implied that an fNIRS individual analysis of eight samples is not sufficient to analyze between-group effects. And the analysis of other brain regions and further fNIRS study may be based on the results of this study.

It is noteworthy that only case S3, which was an infarction of pons, differed from the other cases. On the basis of neuroanatomy, we understand that cerebral infarction in both pons and hemispheres could cause corticospinal tract injury which results in hemiplegia. The degree of corticospinal tract injury is related to the recovery of motor function (52).One mechanism of motor recovery for patients with pontine infarct is that the peri-infarct areas compensate for corticospinal tract damage at the pons (53). However, the blood flow change around the pons is too deep to be detected by fNIRS detectors; a different hemodynamic change on cortex may be detected. In fact, the activation channel analysis of S3 is indeed different from other cases. The activation analysis of S3 showed the maximum mean beta value located in the right premotor cortex. Deactivations of left sensorimotor cortex, left premotor cortex, and bilateral prefrontal cortex were observed. In addition, it is reported that the reorganization around subcortical lesions occurs at the level of corona radiata and pons in stroke patients (53, 54).Whether there is any consistency between the reorganization of pons and hemisphere still needs to be discussed; recent studies focused mainly on the functional connection (resting state) of cerebral cortical blood flow and pons by MRI studies (55). fNIRS studies on the mechanism of cerebral blood flow changes and accompanying functional recovery after pontine infarction are still scarce, and could potentially be our next research direction.

### Clinical outcomes

In our study, we used clinical assessment to assess recovering motor functions of the upper extremity during a grip-release task in persons with stroke. These scales were identifying gross and fine motor performance for persons with stroke. All eight participants showed improvements after intervention for the ARAT, FMA-arm, and grip strength scores. But whether the effectiveness of the dynamic hand orthosis in the early subacute stroke period would rely on RCT study will be discussed in another paper (15).

Grip strength is easy to measure and less time-consuming than arm muscle strength measurements, and grip strength can be a representative measure of muscle weakness of the entire upper extremity after stroke (56).Although the motor task designed varied in previous experimental designs, such as raising arms, at the early stage of stroke difficulties were reported as a negative aspect for those with proximal weakness, shoulder subluxation, shoulder pain, or shoulder hand syndrome, so patients could not complete these movements continuously, which made it very difficult to use fNIRS to evaluate upper limb movements in the subacute period. Considering the subject's low grip strength, the fNIRS measurement task may be challenging, but following the inclusion criteria, we selected patients who had partial active finger movement so as to ensure this completion of the grasp-release ring task. All the eight subjects completed the experimental design and clinical test scores improved after intervention, but fNIRS activation analysis did not show more novel results. One possible reason for this is that fNIRS is more sensitive when evaluating gross motor function and less sensitive to fine motor function of the hand in the early subacute stroke recovery. Thus, we could infer that this grasp-release ring task was not appropriate to achieve our fNIRS research objective and a more sensitive motor task or more sensitive evaluating indicator should be used in further studies.

In general, it is hoped that this fNIRS case series study of dynamic hand orthosis combined with unilateral task-oriented training in subacute stroke will provide clinicians more help to design and observe the recovery process of individuals. In the future, it would strengthen the results to follow a group of participants in an extended rehabilitation training time of 6–12 weeks, 3–6 months post-stroke, or try designs which bring stronger hemodynamic changes to determine persistent effect.

### Limitations and future directions

There is no uniform pattern or standard for the tasks used in fNIRS studies. Studies have shown that hemodynamic responses can be regulated by frequency (57, 58), intensity (59, 60), and complexity of tasks or stimulus (37). In our study, each subject tried to complete the unified grasping-release ring task with the same frequency, but the effects with different frequencies and intensity on brain activation could not be collected. It has been reported that head motion can affect the results of cerebral blood flow measured by fNIRS. In recent research, dense fNIRS probes were set to obtain more precise information about task-related neural signals, and motion artifacts were corrected using a technique based on moving standard deviation and spline interpolation or an applied use of smartphones (61, 62). In our design, the participants' head movements were strictly suppressed during the whole task using a chinrest, and the light probes were fixed through an integral plastic sheet to deal with motion artifacts. However, completely filtering noise from neural signals remains a challenge. Skin blood flow has been noticed as another possible influence (63, 64). At present, it is generally admitted that the NIRS-Hb signal mainly reflects task-related hemodynamic changes in the gray matter, so care must be taken when comparing the NIRS-Hb signal with the extracranial blood flow. In some studies, short-separation channels that measured hemodynamic signals from extracranial tissues were used; the blood flow difference between long and short channels could distinguish regional cortical blood flow or scalp blood flow (62).In verbal fluency tasks or cognitive tasks, skin blood flow must be considered because that would induce sympathetic hyperactivation. In our study, the influence of this factor was not distinguished. Sensitive design and sufficient sample size are challenges in future studies.

### Conclusions

In this case series, we have assessed the activation change of bilateral SMC, PFC, and PMC in eight early subacute right hemiplegia stroke patients with unilateral task-oriented therapy and a hand orthosis by fNIRS. Left premotor cortex, left sensorimotor cortex, and right supplementary motor cortex may be the primary regions of interest. But considering the limitations of the discussion, a grasp-release ring task was not appropriate to achieve our fNIRS research objective and a more sensitive motor task and evaluation indexes need to be used to discuss mechanisms from rehabilitation interventions aimed at improving upper extremity motor function.

## Data availability statement

The original contributions presented in the study are included in the article/Supplementary material, further inquiries can be directed to the corresponding author.

## Ethics statement

The studies involving human participants were reviewed and approved by Regional Committee for Medical and Health Research Ethics (No.2017/1915 REK sør-øst D) and the China Rehabilitation Research Center Ethics Committee (No.CRRC-IEC-RF-SC-005-01). The patients/participants provided their written informed consent to participate in this study.

## Author contributions

CL is the first author and wrote the paper. TZ is the corresponding author. YW and CL implemented and collected data. TZ, BL, and XD designed the experiment. FH was the occupational therapy guide and explained fNIRS methodology. HZ was the clinical scale tester. All authors discussed results, article, and approved the submitted version.

## Funding

Support for this study was provided by Funding-China Rehabilitation Research Center Funding (2020-Q4, 2020-10, 2019zx-01), National Key R&D Program of China (2020YFC2004105) and Sunnaastiftelsen.

## Conflict of interest

The authors declare that the research was conducted in the absence of any commercial or financial relationships that could be construed as a potential conflict of interest.

## Publisher's note

All claims expressed in this article are solely those of the authors and do not necessarily represent those of their affiliated organizations, or those of the publisher, the editors and the reviewers. Any product that may be evaluated in this article, or claim that may be made by its manufacturer, is not guaranteed or endorsed by the publisher.

## Abbreviations

SMC: Sensorimotor cortex
PMC: Premotor cortex
PFC: Prefrontal cortex
UL group: Unilateral task-oriented training group
ARAT: Action research arm test
FMA-arm: the Fugl-Meyer Assessment Upper Extremity Scale
FNIRS: Functional near infrared spectroscopy
HbO: Oxygenated hemoglobin
ROI: Region of interest
Ipsi-hemisphere: Ipsilesional hemisphere
Contra-hemisphere: Contralesional hemisphere.
