# Supplementary figures and images for: A study of dynamic hand orthosis combined with unilateral task-oriented training in subacute stroke: A functional near-infrared spectroscopy case series
Source: Front Neurol. 2022 Aug 11;13:907186. doi: 10.3389/fneur.2022.907186 (PMC9410701; doi:10.3389/fneur.2022.907186)

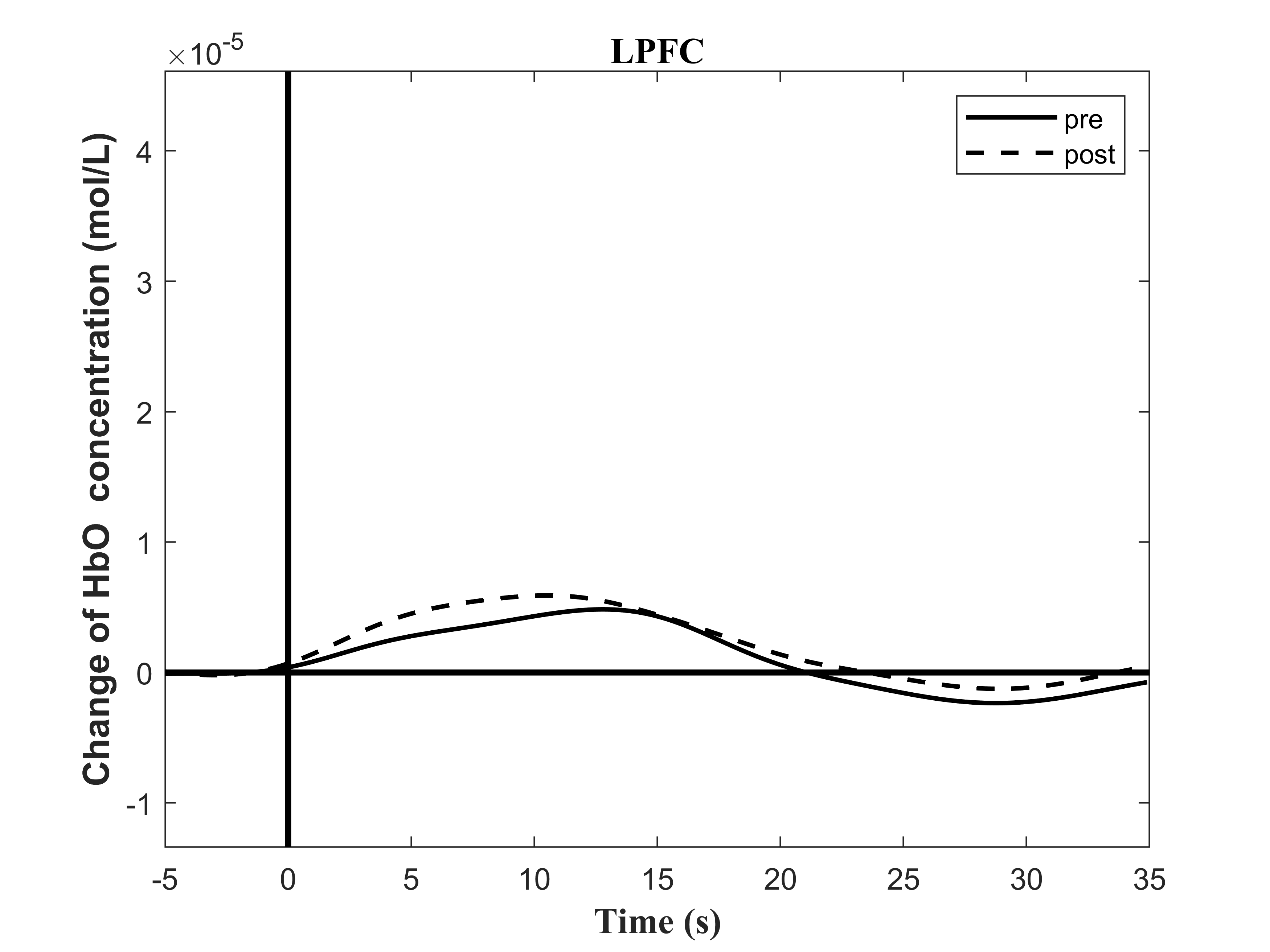

Supplement: Supplementary file 1 [file Image_1.TIF]

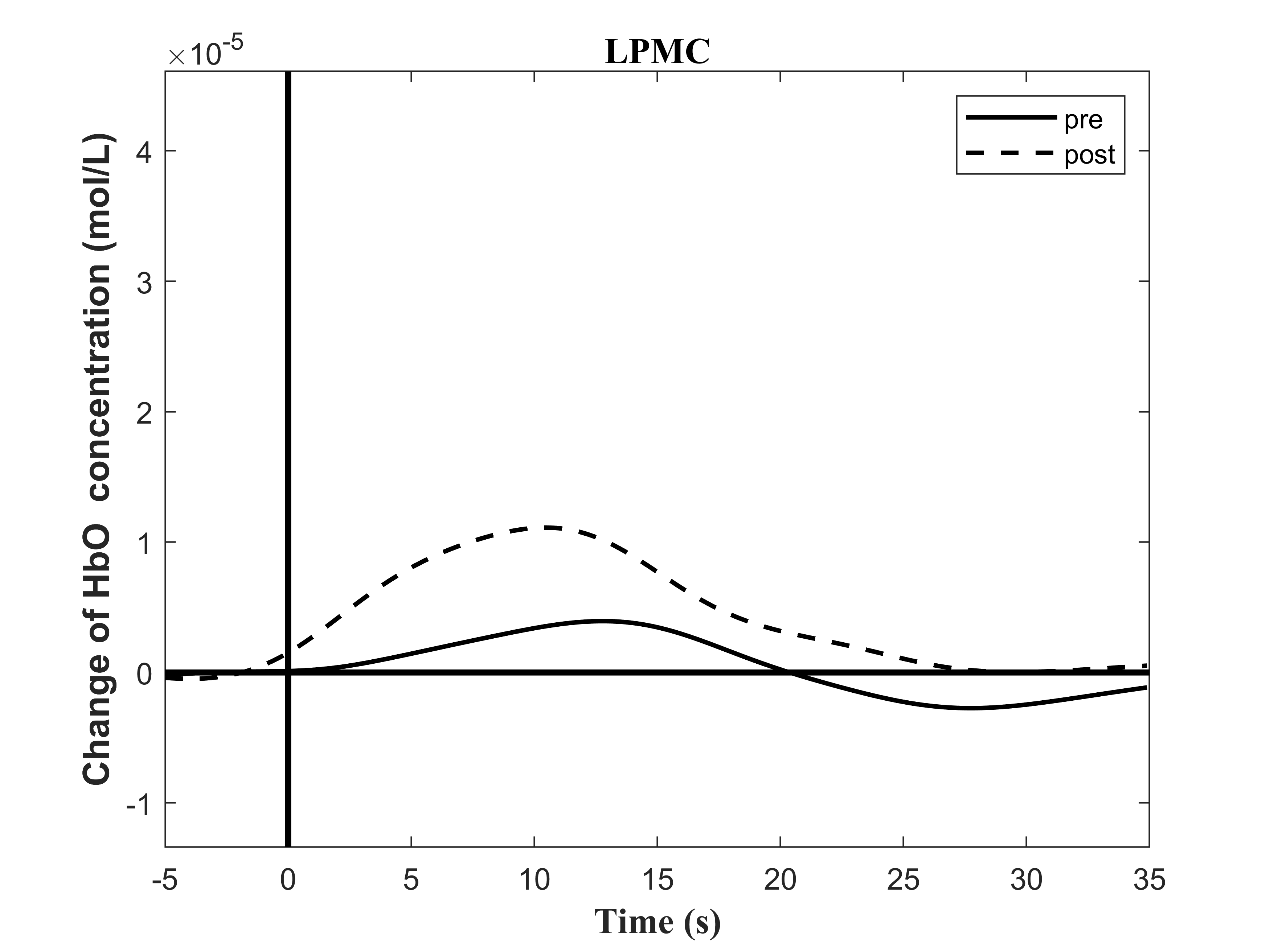

Supplement: Supplementary file 2 [file Image_2.TIF]

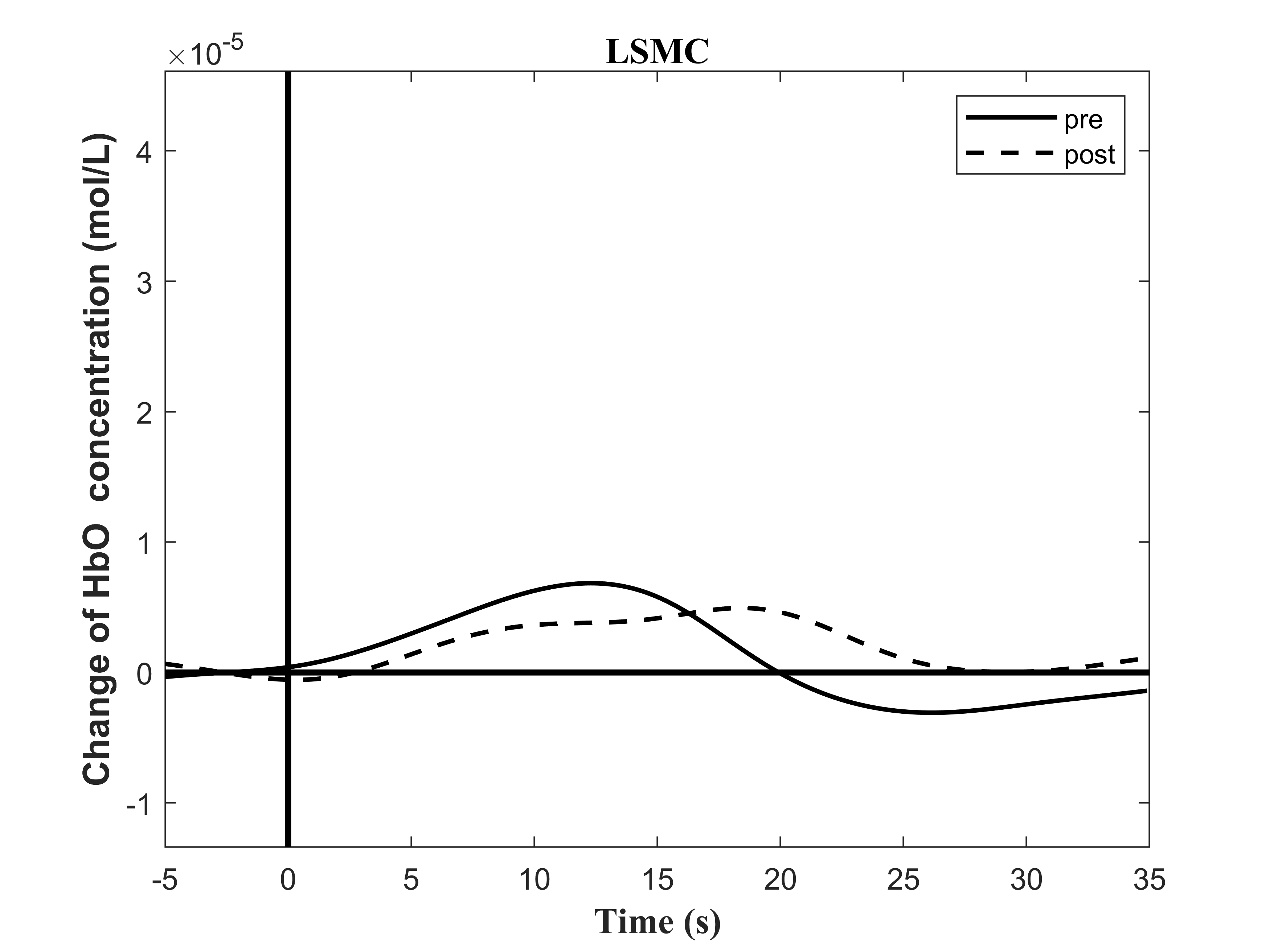

Supplement: Supplementary file 3 [file Image_3.TIF]

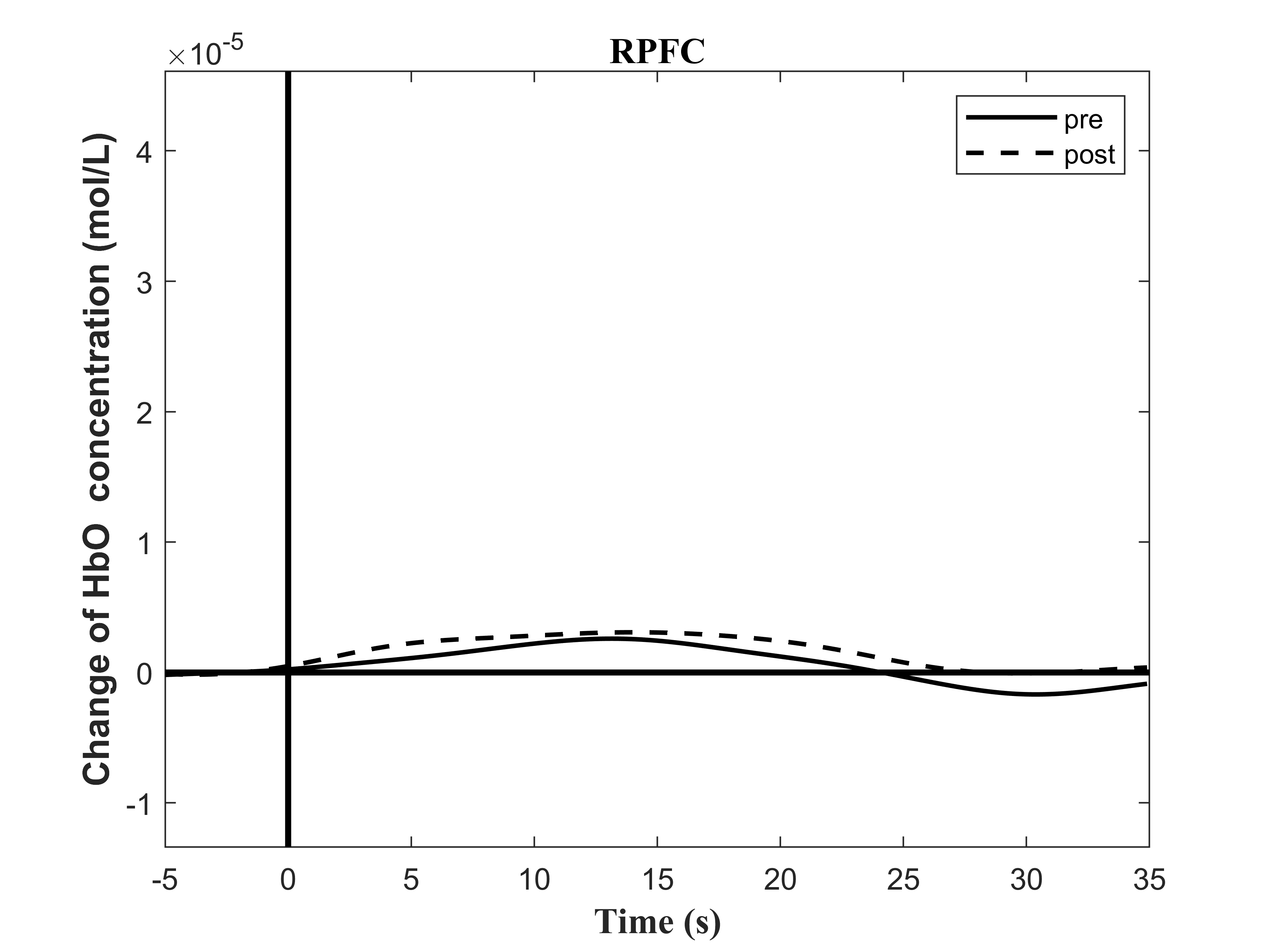

Supplement: Supplementary file 4 [file Image_4.TIF]

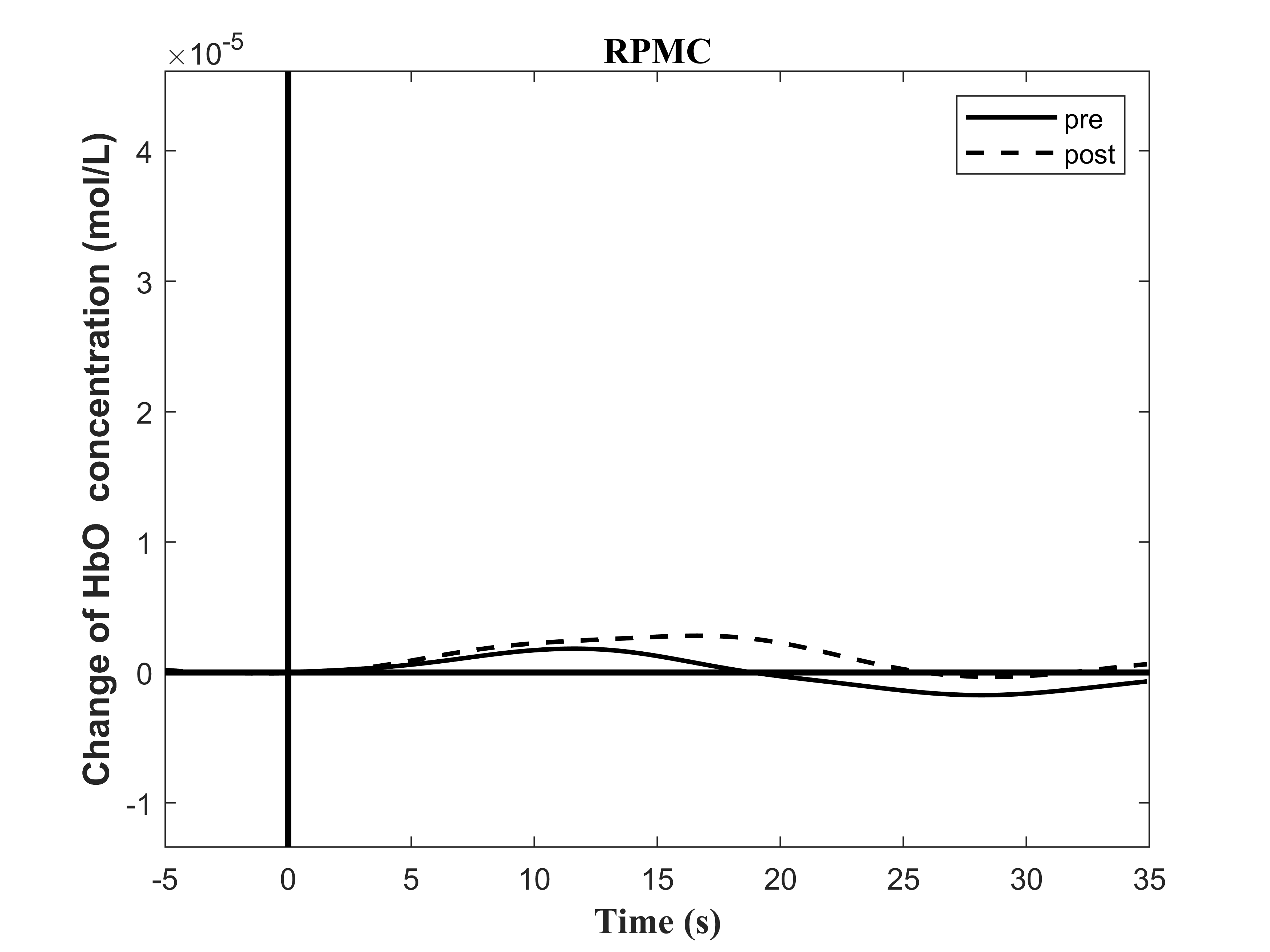

Supplement: Supplementary file 5 [file Image_5.TIF]

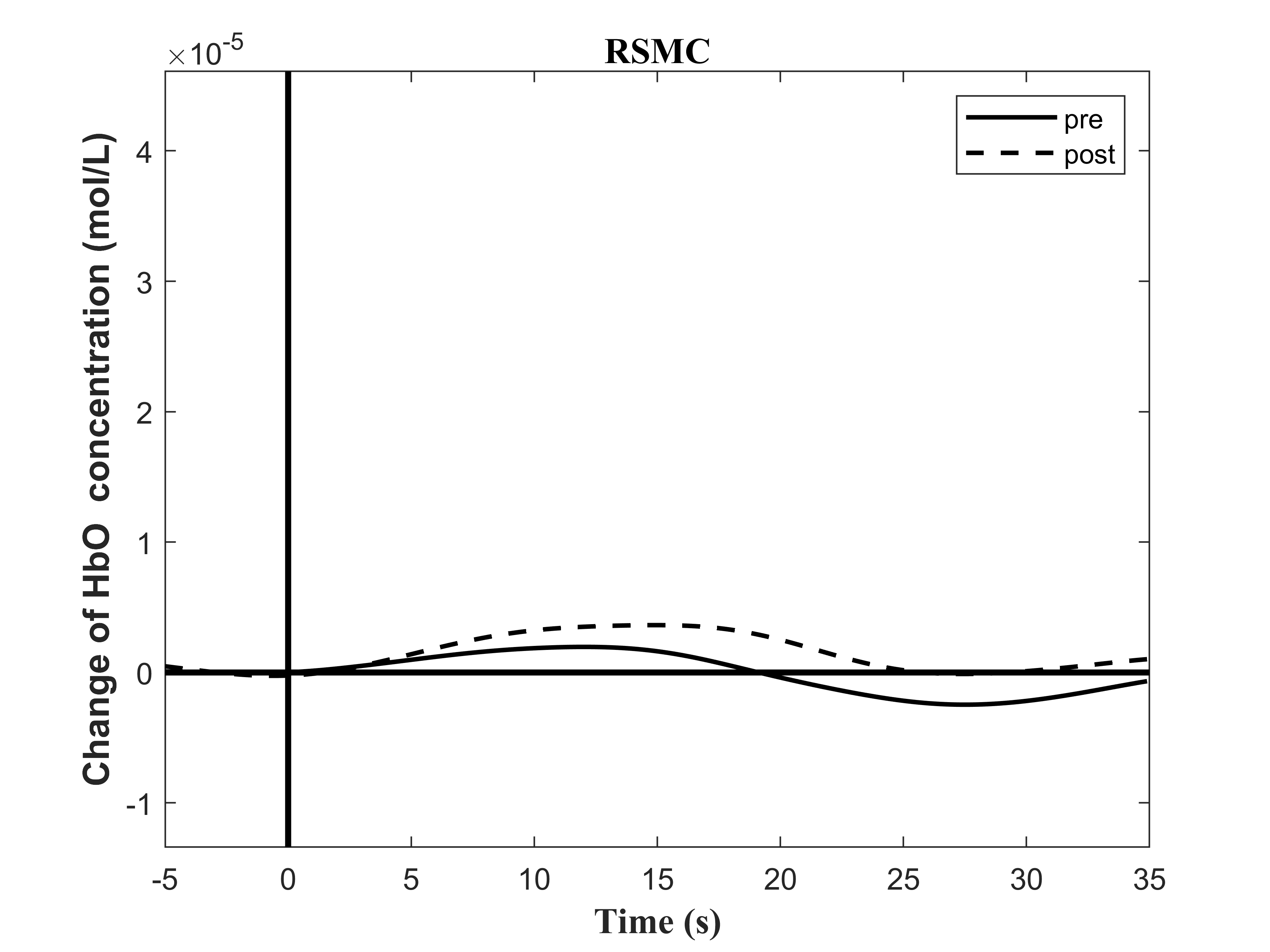

Supplement: Supplementary file 6 [file Image_6.TIF]
